# Body Image Misperceptions Among Tunisian Adolescents

**DOI:** 10.7759/cureus.48981

**Published:** 2023-11-17

**Authors:** Mohamed Ferhi, Amira Dalhoumi, Rim Ghammam, Jihenne Mannai

**Affiliations:** 1 Psychiatry, Ibn El Jazzar University Hospital, Kairouan, TUN; 2 Epidemiology and Public Health, Farhat Hached University Hospital, Sousse, TUN

**Keywords:** adolescence, socio-economic factors, weight status, obesity, body image distortion

## Abstract

Background

Obesity is a globally recognized health concern with profound consequences for individual health, especially among adolescents. Tunisia, like many countries, is experiencing alarming rates of adolescent obesity. Understanding adolescents' perceptions of their weight status and the factors influencing these perceptions is pivotal for developing targeted interventions and policies to counteract the rising obesity trends.

Objective

To determine the accuracy of weight status perceptions among Tunisian adolescents compared to objective metrics and to identify sociodemographic characteristics associated with the inaccurate estimation of weight status.

Methods

A cross-sectional, school-based study was conducted among adolescents attending secondary schools in Sousse, Tunisia, during the 2017-2018 academic year. A total of 1399 students participated, with anthropometric measurements taken, and a pre-tested Arabic questionnaire administered to gather sociodemographic data and perceived weight status, assessed using the Figure Rating Scale (FRS). The accuracy of perceived weight status was determined by comparing the measured weight status with participants' self-reported perceptions.

Results

The study achieved an 86.68% response rate, with over half of the participants being female (60.5%), and the average age being 17 years. The majority of adolescents (41%) perceived themselves as having normal body weight, while 34.5% perceived themselves as underweight, 16.6% as overweight, and 7.9% as obese. However, based on BMI categories, 72.6% had a normal measured weight, 20.4% were overweight, and 6.9% were obese. A substantial proportion of participants (45.6%) underestimated their weight status, with a significant proportion being objectively overweight or obese (26%). Furthermore, we found a significant association between the perception of weight accuracy and both gender (p = 0.010) and the mother's educational level (p = 0.035).

Conclusions

The findings revealed a disparity between perceived and actual weight status among Tunisian adolescents, with a significant underestimation of weight status, particularly among those who are overweight or obese. The results highlighted the crucial need for interventions that address weight perception inaccuracies and promote healthy weight awareness and management among adolescents in Tunisia.

## Introduction

Obesity, a major health concern globally, has significant implications for individual health, being a potent risk factor for various chronic conditions like cardiovascular diseases, endocrine derangements (diabetes and thyroid disease), musculoskeletal disorders, and certain cancers [1]. The increasing obesity rates underscore the urgent need for enhanced awareness and effective prevention strategies [1].

In the context of the Eastern Mediterranean Region, alarming obesity rates range from 7% to 45% [2]. Importantly, obesity's origins often manifest in adolescence, a phase strongly predictive of adult obesity [3], highlighting adolescence as a crucial intervention point.

In Arab countries, adolescent obesity is at a crisis point, driven by factors like nutritional transitions, urbanization, and sedentariness [4]. Among these nations, Tunisia has witnessed a significant rise in adolescent overweight and obesity. Between 1996 and 2005, Tunisia experienced a 1.5 to 5.0-fold increase in prevalence rates for overweight among adolescent girls and boys, respectively [5]. This concerning trend persists, with current rates indicating that 11.6% to 48.9% are overweight, and 2.7% to 10.0% are obese [5,6].

Changes in health behaviors are more likely to be successful when the individual recognizes there is a problem [7]. Moreover, these behavioral modifications are often more effectively managed and sustained within a medical environment, where professional guidance and support are readily available. Self-awareness regarding one's weight often fuels the motivation to maintain a healthy weight. Conversely, misperceptions can diminish the motivation for weight regulation [8].

Body image is a psychosocial dimension, defined by Schilder in the 1930s as the picture of our own body which we form in our mind [9]. Cultural and societal norms dictate body image ideals, often favoring thinness for females and muscularity for males [4,10]. Societal pressures and media representations may lead some, predominantly females, to perceive themselves as overweight, leading to different health challenges [4,10]. Additionally, external factors, like parents’ beliefs, play significant roles in shaping these perceptions [11]. These factors mean that interventions promoting healthy weight need to understand the differences between adolescents to ensure accurate targeting of messages.

Given these intricacies in weight perception and the knowledge gaps in Tunisian adolescents' body image perceptions, this cross-sectional study aimed to determine the accuracy of their weight status perceptions compared to objective metrics and to identify sociodemographic characteristics associated with the underestimation of weight status.

## Materials and methods

Study design

This was a cross-sectional, school-based study conducted among adolescents attending secondary schools in the governorate of Sousse, Tunisia, during the 2017-2018 academic year. This research was part of a broader study undertaken during the same timeframe, aiming to identify psychological factors and addictive behaviors (such as smoking and video gaming) related to body weight perception within this population. In Tunisia, the secondary school system comprises the common core (first and second years) and terminal studies (third and fourth years). Upon completing the fourth year, students are required to sit for a national examination, successful completion of which qualifies them for higher education.

Target population

Initially, we obtained a list of all public secondary schools in Sousse City. Of these, 10 out of 12 schools met the eligibility criteria for the study, having an enrollment of 500 students or more. Subsequently, four secondary schools were randomly chosen from this eligible pool to achieve the desired sample size. The classes within these selected schools were then stratified by grade level (first through fourth year). From each grade, specific classes were chosen at random. In total, 59 classes were selected across the schools to meet the sample size requirements. Only students from these selected classes who gave their consent were included in the study. Necessary permissions to approach the schools in the study were secured from the Regional Directory of Education, the Tunisian Ministry of Education, and the respective school authorities.

Sample size

To determine the necessary sample size, we utilized the Epi Info™ version 6 software (Centers for Disease Control and Prevention, Atlanta, GA, USA). Multiple sample size calculations were performed based on the prevalence of various factors identified in the primary study (e.g., sedentariness, obesity, gaming addiction). The highest sample size was derived from the 26% smoking prevalence among Tunisian adolescents [12]. With an alpha (type I error) set at 5%, a precision level of 4.5%, and a cluster effect of 2, the calculated sample size was 1095 students. To account for potential missing data, we increased this number by 20%, resulting in a minimum required participant count of 1314 adolescents. Finally, we were able to include 1399 students.

Anthropometric measurements

Prior to data collection, the research team explained the study purpose while simultaneously dispelling any doubts students might have regarding their data confidentiality and anonymity. Participants' height and weight were measured by trained interviewers upon receiving consent. Parents or legal guardians provided written informed consent for all participants under the age of 18. Heights were recorded in centimeters (cm) to an accuracy of 0.5 cm using a wall-mounted measuring scale. Weights were measured in kilograms (kg) with a pre-calibrated portable digital scale (Tanita, Tanita Corporation of America, Inc., Arlington Heights, IL), accurate to 0.1 kg. All measurements were taken with students in light clothing and without shoes.

Body mass index (BMI) was calculated as the body weight (in kg) divided by the square of the height (in m^2^). The International Obesity Task Force's age and gender-specific BMI cut-off points [13] were used to classify participants into four weight categories: underweight, normal weight, overweight, and obese.

Self-reported questionnaire

A pre-tested Arabic questionnaire was self-administered in classrooms by trained investigators. The questionnaire was designed to gather the following data:

Socio-demographic characteristics such as age, gender, level of education, parents' level of education, and parents' professional status. The level of education was considered high for university education, medium for secondary school education, and low for primary school education or illiteracy.

**Figure 1:**
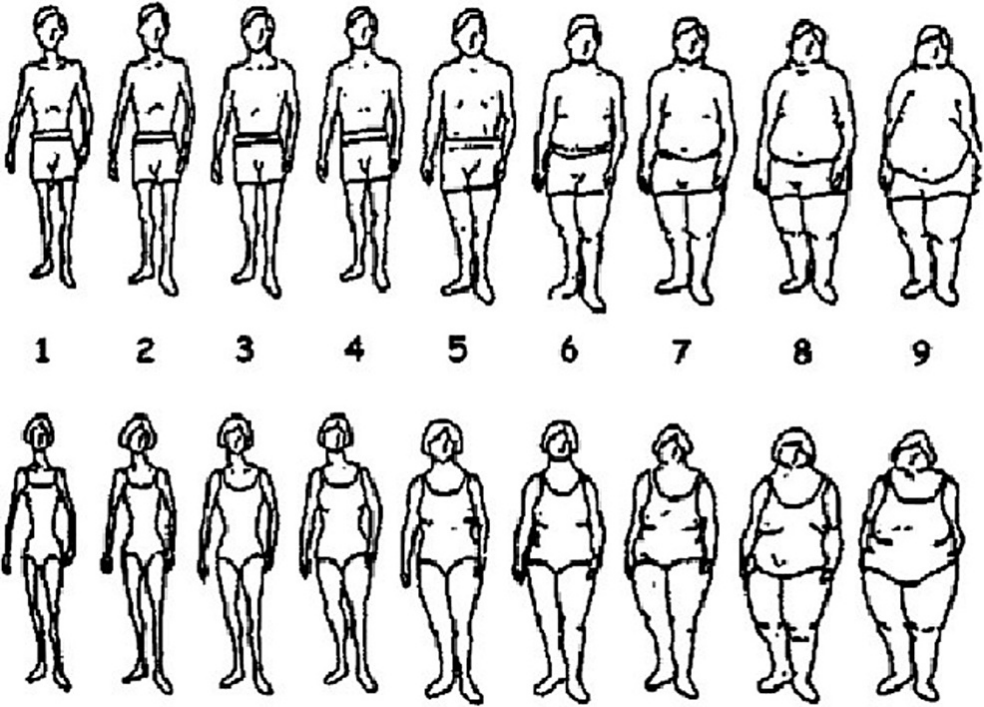
The Figure Rating Scale Image from Stunkard et al. [14]

Perceived weight status was assessed using the Figure Rating Scale (FRS), which was developed by Stunkard et al. [14]. This tool has demonstrated reliability across various populations, including those in the Middle East [15], and has been validated for use in children and adolescents aged 3-18 years [16]. The FRS comprises nine male and nine female silhouette images (Figure 1), representing body sizes from extremely thin (labeled “1”) to morbidly obese (labeled "9”). These images correspond to four perceived weight categories: 1 and 2 for underweight; 3 and 4 for normal weight; 5-7 for overweight; and 8 and 9 for obesity [17]. Participants were instructed to select the silhouette that they felt most closely matched their own body image.

Outcome measure: Accuracy of perceived weight status

The primary outcome was the accuracy of participants' perceived weight status. This was derived by comparing the measured weight status (based on BMI categories) with the participants' self-reported perceptions. Participants were subsequently categorized as “underestimators”, “overestimators”, or “accurate estimators”.

Ethical considerations

This study was conducted with strict adherence to laws and with respect for individual integrity. The protocol, questionnaire, and consent form received approval from the ethics committee of Farhat Hached University Hospital in Sousse on September 5, 2017. In addition to the aforementioned authorization, free, informed, and written consent was obtained from the parents of the students prior to data collection. Students were granted full autonomy to decide whether to participate or decline. Our study posed no risk to the participants in any form.

Statistical analysis

Our primary hypothesis was that there would be a significant discrepancy between adolescents' self-perceived weight status and their actual weight status based on BMI measurements. We anticipated that a substantial proportion of adolescents would underestimate their weight status. Another key hypothesis was that sociodemographic factors, particularly gender and parents' educational level, would significantly influence the accuracy of weight status perception among adolescents.

Data input and analysis were conducted using the Statistical Package for the Social Sciences (IBM SPSS Statistics for Windows, IBM Corp., Version 20.0, Armonk, NY). Descriptive analysis was carried out for all participant demographic, socio-economic, and prevalence data. Qualitative variables were presented as counts (n) and percentages (%). Quantitative variables were expressed as the mean (M) accompanied by its standard deviation (±SD). For comparisons, the Chi-square test (χ2) was employed for percentages, and a two-tailed Student's t-test was used for comparing means on independent samples, after ensuring the assumptions of normality and homogeneity of variances were met. The significance threshold (p) was established at 5%, meaning that a p-value less than 0.05 was considered statistically significant.

## Results

Sociodemographic results

Table 1 presents the sociodemographic characteristics of the sample participants. The study involved four secondary schools, with a total of 1399 students participating, achieving a response rate of 86.68%. Over half of the sample was female (60.5%) with a male/female sex ratio of 0.65 and the average age was 17 years. The four grades of secondary education were evenly represented in our study, each accounting for approximately 25%. Among the participants, 29.1% had repeated at least one year during their studies. The majority of parents of participating students had completed secondary or higher education.

**Table 1:** Sociodemographic characteristics of the study participants (N=1399) Notes: totals vary by sociodemographic variables due to missing data; n = counts; % = percentages. ^a^ The response rates for each individual school. ^b^ The level of education includes illiterate or primary school (low), secondary school (intermediate), and university (high).

	n	%
Gender		
Male	553	39.5
Female	846	60.5
Age categories, in years		
[14-16]	535	38.2
(16-17]	346	24.8
>17	518	37.0
Response rate by school ^a ^		
Ibn Rochd	80.4	
Ahmed Noureddine	88.1	
Lycée Pilote	92.3	
Abdelaziz El Behi	85.2	
Overall response rate	86.6	
Distribution by school		
Ibn Rochd	317	22.7
Ahmed Noureddine	436	31.2
Lycée Pilote	353	25.2
Abdelaziz El Behi	293	20.9
Grade		
1st year	368	26.3
2nd year	304	21.7
3rd year	354	25.3
4th year	373	26.7
Section		
Common core	366	26.2
Natural sciences	312	22.3
Literature studies	193	13.8
Computing	87	6.2
Mathematics	119	8.5
Economy	209	14.9
Technology	113	8.1
Repeating a grade		
Yes	407	29.1
No	992	70.9
Mother’s level of education^b^, n=1381		
Low	388	28.1
Intermediate	489	35.4
High	504	36.5
Father’s level of education^b^, n=1381		
Low	326	23.6
Intermediate	480	34.8
High	575	41.6
Mother’s occupation, n=1363		
No occupation	683	50.1
Factory worker	121	8.9
Private sector	106	7.8
Public sector	328	24.1
Executive	125	9.1
Father’s occupation, n=1297		
No occupation	81	6.2
Factory worker	211	16.3
Private sector	377	29.1
Public sector	399	30.8
Executive	299	17.6

Perceived and measured body weight

**Figure 2:**
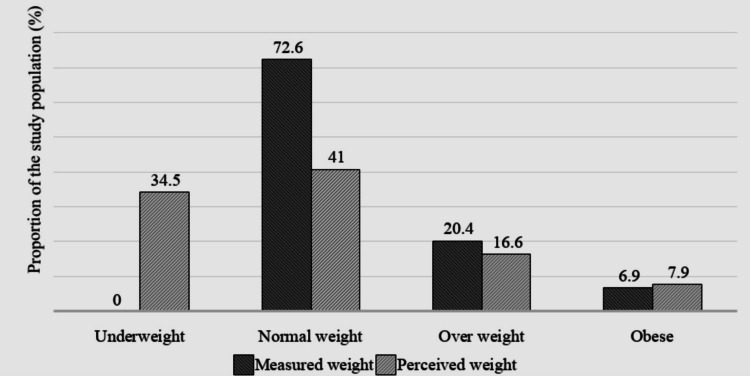
Proportion of measured and perceived body weight status of the study participants (N=1399)

According to body image, a significant proportion of adolescents perceived themselves as underweight (34.5%), overweight (16.6%), or obese (7.9%), while the majority, accounting for 41%, perceived themselves as having a normal body weight. Regarding measured body weight, 72.6% had a normal measured weight, 20.4% were overweight, and 6.9% were obese (Figure 2).

Table 2 presents the associations between weight perception status and adolescents' sociodemographic characteristics, as well as their measured weight status. More males than females perceived their weight status to be within an acceptable weight range (45.0% vs 38.4%, p = 0.089). Parents’ level of education was significantly associated with adolescents’ perceived weight status. Adolescents with highly educated mothers perceived themselves as obese in 6.6% of cases and overweight in 20.3% of cases. Within participants whose mothers had an intermediate educational level, we found a percentage of 9.2% and 17.4% for perceived obesity and overweight respectively. Among the group with illiterate or primarily educated mothers, the percentage of perceived obesity and overweight were 7.8% and 10.4% respectively. In fact, the mother’s level of education was significantly associated with the perceived weight status of their children (p < 0.001). A significant association of the perceived weight was also found with the father’s level of education (p = 0.005).

**Table 2:** Association between perceived weight status, measured weight status, and sociodemographic characteristics among study participants Notes: totals vary by sociodemographic variables due to missing data; n = counts; % = percentages; SD = standard deviation. ^a^ The percentage of counts out of the overall of the corresponding row. ^b^ The level of education includes illiterate or primary school (low), secondary school (intermediate), and university (high).

	Perceived weight, n (%) ^a ^					P-Value
	Underweight	Normal weight	Overweight	Obese	Overall	
Measured weight status					1386	<0.001
Normal weight	468 (46.4)	444 (44.0)	76 (7.5)	20 (2.0)	1008	
overweight	8 (2.8)	116 (41.3)	124 (44.1)	33 (11.7)	281	
obese	2 (2.1)	8 (8.2)	30 (30.9)	57 (58.8)	97	
Age, mean (SD)	17.1 (1.5)	16.9 (1.5)	16.9 (1.4)	17.2 (1.4)		0.123
Gender					1386	0.089
Male	177 (32.4)	246 (45.0)	87 (15.9)	37 (6.8)	547	
Female	301 (35.9)	322 (38.4)	143 (17.0)	73 (8.7)	839	
Age category					1386	0.431
[14-16]	173 (32.6)	230 (43.3)	91 (17.1)	37 (7.0)	531	
[16-17]	121 (35.5)	139 (40.8)	58 (17.0)	23 (6.7)	341	
>17	184 (35.8)	199 (38.7)	81 (15.8)	50 (9.7)	514	
Grade					1386	0.101
1st year	107 (29.4)	166 (45.6)	62 (17.0)	29 (8.0)	364	
2nd year	117 (38.6)	121 (39.9)	47 (15.5)	18 (5.9)	303	
3rd year	127 (36.5)	134 (38.5)	64 (18.4)	23 (6.6)	348	
4th year	127 (34.2)	147 (39.6)	57 (15.4)	40 (10.8)	371	
Repeating a grade					1386	0.150
Yes	143 (35.7)	157 (39.2)	60 (15.0)	41 (10.2)	401	
No	335 (34.0)	411 (41.7)	170 (17.3)	69 (7.0)	985	
Mother’s level of education ^b ^					1375	<0.001
Low	154 (40.1)	160 (41.7)	40 (10.4)	30 (7.8)	384	
Intermediate	187 (38.2)	172 (35.2)	85 (17.4)	45 (9.2)	489	
High	134 (26.7)	233 (46.4)	102 (20.3)	33 (6.6)	502	
Father’s level of education ^b^					1374	0.005
Low	118 (36.2)	125 (38.3)	47 (14.4)	36 (11.0)	326	
Intermediate	183 (38.4)	183 (38.4)	73 (15.3)	38 (8.0)	477	
High	172 (30.1)	258 (45.2)	106 (18.6)	35 (6.1)	571	
Mother’s occupation					1373	0.089
No occupation	265 (38.0)	270 (38.7)	107 (15.4)	55 (7.9)	697	
Factory worker	45 (37.8)	45 (37.8)	18 (15.1)	11 (9.2)	119	
Private practice	31 (29.2)	46 (43.4)	19 (17.9)	10 (9.4)	106	
Public sector or an executive	129 (28.6)	204 (45.2)	86 (19.1)	32 (7.1)	451	
Father’s occupation					1287	0.555
No occupation	23 (29.1)	34 (43.0)	14 (17.7)	8 (10.1)	79	
Factory worker	72 (34.3)	91 (43.3)	29 (13.8)	18 (8.6)	210	
Private practice	137 (36.6)	139 (37.2)	72 (19.3)	26 (7.0)	374	
Public sector or an executive	207 (33.1)	271 (43.4)	100 (16.0)	46 (7.4)	624	

We found significant differences between measured and perceived weight status within our study population. In the case of objectively measured obesity, 58.8% of the adolescents accurately perceived their weight status, while 41.2% underestimated it. Regarding measured overweight status, only 44.1% estimated their weight correctly (Table 2).

Figure 2 illustrates that nearly all participants (97.9%) who perceived themselves as underweight had a normal body weight according to BMI. About one-third of the participants (33%) who believed they were overweight actually had a normal body weight, while nearly half of those who perceived themselves as obese (48.1%) had a measured weight status of normal or overweight.

Accuracy of body weight perception

**Figure 3:**
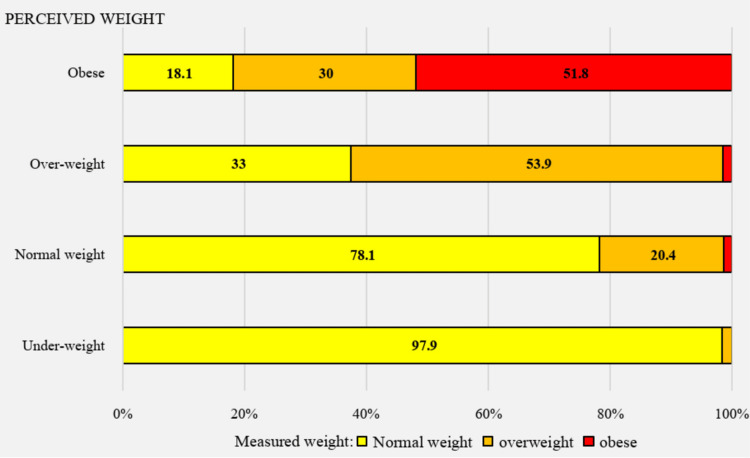
Measured weight status, by perceived weight status (n=1386)

**Figure 4:**
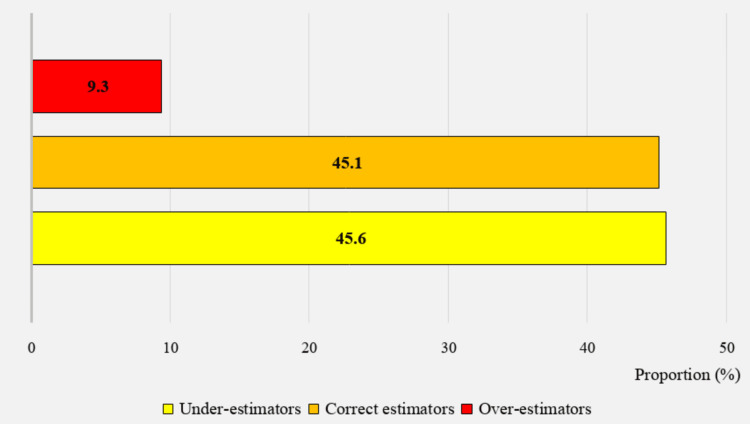
Proportion of the accuracy of perceived body weight (n=1386)

Results indicate that the largest percentage of the study population (45.6%) underestimated their weight status, while 45.1% estimated it accurately, and 9.3% overestimated it (Figure 3). Figure 4 shows that slightly more than one-fourth of those in the underestimators' category (26%) had objectively measured obesity or overweight, while the remaining 74% had a BMI within the normal range. Among the 9.3% of the population who overestimated their weight status, the majority (74%) had a measured BMI within the normal range, while 26% had a BMI above that range.

**Figure 5:**
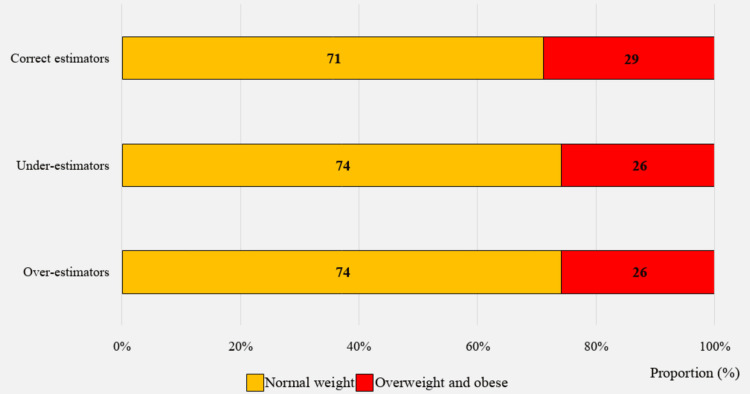
Association between the accuracy of body weight perception and the measured weight (n=1386)

**Figure 6:**
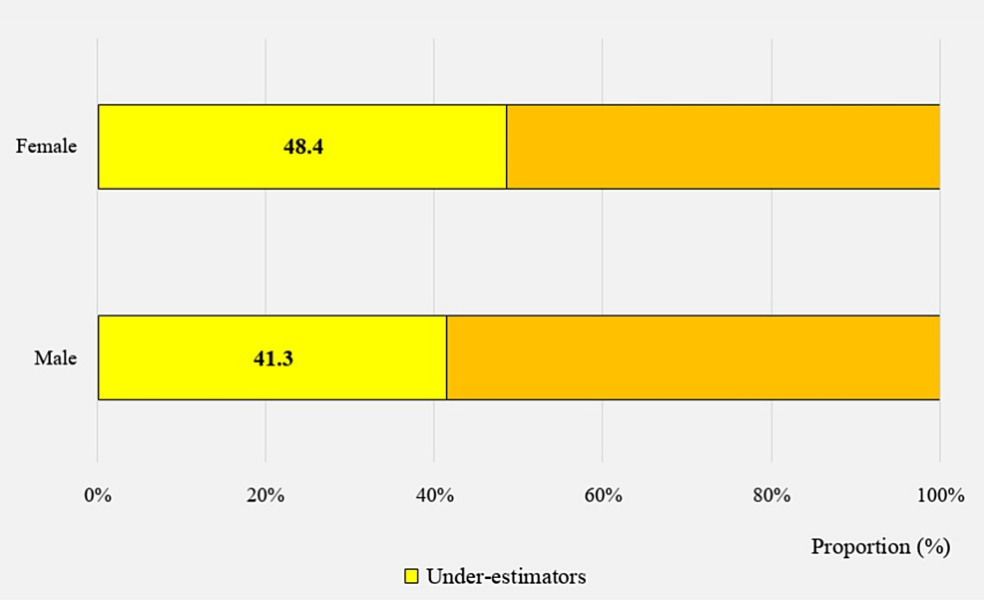
Proportion of underestimators, by sex (n=1386)

Table 3 shows the associations between the accuracy of body weight perception and the sociodemographic characteristics among study participants. The underestimation of body weight was uniformly distributed across age intervals, with approximately 45% in each interval (Figure 5). However, this varied considerably by sex, as we observed a higher underestimation rate among girls (p = 0.01). In fact, 41.3% of boys and 48.4% of girls underestimated their own weight category (Figure 6).

**Table 3:** Association between the accuracy of body weight perception and sociodemographic characteristics among study participants Notes: totals vary by sociodemographic variables due to missing data; n = counts; % = percentages. ^a^ The percentage of counts out of the overall of the corresponding row. ^b^ The level of education includes illiterate or primary school (low), secondary school (intermediate), and university (high).

	Underestimators, n (%) ^a^	Normal and overestimators, n (%) ^a ^	P-Value
Gender, n=1386			0.010
Male	226 (41.3)	321 (58.7)	
Female	406 (48.4)	433 (51.6)	
Age category, n=1386			0.984
[14-16]	241 (45.4)	290 (54.6)	
(16-17]	155 (45.5)	186 (54.5)	
>17	236 (45.9)	278 (54.1)	
Grade, n=1386			0.480
1st year	158 (43.4)	206 (56.6)	
2nd year	149 (49.2)	154 (50.8)	
3rd year	160 (46.0)	188 (54.0)	
4th year	165 (44.5)	206 (55.5)	
Repeating a grade, n=1386			0.156
Yes	186 (46.4)	215 (53.6)	
No	446 (45.3)	539 (54.7)	
Mother’s level of education^b^, n=1375			0.035
Low	189 (49.2)	195 (50.8)	
Intermediate	207 (41.2)	295 (58.8)	
High	233 (47.6)	256 (52.4)	
Father’s level of education^b^, n=1374			0.090
Low	147 (45.1)	179 (54.9)	
Intermediate	244 (42.7)	327 (57.3)	
High	236 (49.5)	241 (50.5)	
Mother’s occupation, n=1373			0.055
No occupation	339 (48.6)	358 (51.4)	
Factory worker	55 (46.2)	64 (53.8)	
Private sector	48 (45.3)	58 (54.7)	
Public sector or an executive	182 (40.4)	269 (59.6)	
Father’s occupation	n=1287		0.655
No occupation	34 (43.0)	45 (57.0)	
Factory worker	99 (47.1)	111 (52.9)	
Private sector	175 (46.8)	199 (53.2)	
Public sector or an executive	271 (43.4)	353 (56.6)	

**Figure 7:**
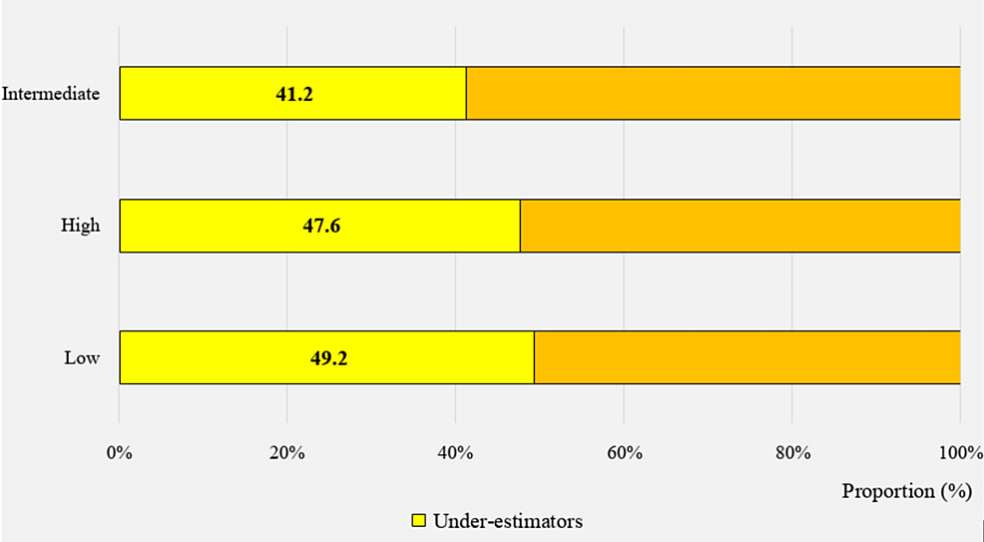
Proportion of underestimators, by the mother's level of education (n=1375)

The accuracy of weight perception was found to be significantly associated with the mother’s level of education. The highest prevalence of underestimators (49.2%) was observed when mothers had the lowest educational level (Figure 7). However, the father’s level of education, as well as the parent’s occupation, did not show a significant association with the accuracy of weight perception, as indicated in Table 3 (p = 0.09, p = 0.055, and p = 0.655, respectively).

## Discussion

As one of the few studies conducted in Tunisia, this original research explored various dimensions of body image among a representative sample of Tunisian adolescents. We employed BMI and FRS to ascertain the accuracy of body weight perception and its correlation with sociodemographic factors.

Our findings showed that 72.6% of the study participants had a normal measured weight, 20.4% were overweight, and 6.9% were obese. In terms of body image, the majority of adolescents, 41%, perceived themselves as having a normal body weight, while 34.5% considered themselves as underweight, 16.6% as overweight, and 7.9% as obese. Remarkably, the largest proportion, almost half of our study population, underestimated their weight status, with over a quarter objectively being overweight or obese. Furthermore, we found a significant association between the perception of weight accuracy and both gender and the mother's educational level.

The global obesity rate among children has experienced a threefold increase from 1975 to 2016 [1]. Specifically, in the Eastern Mediterranean Region, there is a wide-ranging prevalence of overweight and obesity, reaching critical levels in Arab countries [2]. In Tunisia, the prevalence of overweight and obesity among adolescents is a growing concern [18]. According to available data, current prevalence rates for these conditions stand at 11.6% to 48.9% and 2.7% to 10.0%, respectively [5,6]. Our findings align with these statements and are further substantiated by an earlier study conducted in the same region (Sousse), involving 1569 urban school adolescents of corresponding age [19]. Indeed, this obesity epidemic has been associated with a transition to fast food consumption and a lifestyle marked by increased sedentariness and physical inactivity. This pattern, comparable to trends observed in neighboring countries of the Eastern Mediterranean Region, leads to enduring imbalances between energy intake and expenditure [4].

Regarding perceived body weight, a prior study involving 1737 adolescents in the United States revealed that 62% perceived their weight as normal [20]. This raises the question as to why the majority of adolescents perceived themselves within normal weight ranges rather than as overweight or obese, given the substantial proportion of those who underestimated their weight status. Indeed, actual weight status significantly influences body image perceptions among teenagers. Research has shown that perceptions of body weight often tend to be inaccurate when compared to BMI [21]. Therefore, a deeper understanding is needed regarding the development and progression of body size underestimation throughout childhood.

We found that the largest percentage of our study population (45.6%) underestimated their weight status where a significant proportion was objectively overweight or obese (26%). Several studies reported comparable results [6,20,22]. In a study published in 2019 and conducted in Tunisia in the city of Sfax including 1210 school adolescents [6], the prevalence of body weight under-estimation was 37.9%. Additionally, a previous study in Kuwait reported that 50% of obese adolescents considered themselves to be of normal weight [22]. Researchers note an expanding disparity between the reality and perception of body weight status among adolescents [23]. A proposed visual normalization theory suggests this underestimation is relative to visual body size norms, as prevalent larger body sizes recalibrate perceptions of what is considered 'normal', thereby raising the visual threshold for ‘overweight’. Consequently, adolescents develop a misperception of what is a normal and appropriate weight status for their age and height and do not refer to standard measurements and scales to assess their weight and instead tend to compare them to their peers [23].

On the other hand, adolescents may carry the beliefs of their parents and family that being overweight is an indicator of good health and that being overweight will disappear with age, especially among very young teens [11]. In fact, cultural and social norms play a pivotal role in the underestimation of body weight [4,11,22]. Addressing these norms is crucial for effecting a significant change in weight perception. These norms, indeed, may account for the disparities in body weight perception accuracy regarding gender.

As opposed to our study, males tend to underestimate their body weight, whereas females tend to overestimate theirs [20,21,24,25]. In this context, a sub-cohort of 2179 healthy Chinese adolescents randomly selected from schools in Wuhan, China, including 1156 boys and 1023 girls, showed that underestimation was more likely to occur in boys, while overestimation was, conversely, more likely to occur in girls [25]. This discrepancy may be attributed to differing body ideals, with boys associating a muscular physique with a healthy image and girls often associating thinness with beauty and health [4,10]. A study in Malaysia aimed to explore gender variances in BMI, body weight perception, and weight management strategies [24]. A significant number of females (55.7%) preferred an underweight figure as ideal, contrasted by a mere 2% preferring overweight; conversely, more males (30.7%) preferred an overweight figure as ideal, with only 9% preferring underweight [24]. Another study of 6863 Chinese adolescents supported these findings, with girls more frequently viewing themselves as heavier compared to boys [26]. The increased adiposity during puberty may play a role in higher dissatisfaction among girls compared to boys [27].

Nonetheless, some studies agreed with our findings [6,28]. Girls were significantly more likely to have an under-estimated body weight, compared to boys (p = 0.047) in a study conducted in Tunisia including 1210 adolescents [6]. A 2018 nationwide study in Brazil, including 71,740 students aged 12 to 17 from the Study of Cardiovascular Risk in Adolescents (ERICA), revealed that three in ten students showed misperceptions about their body image, with associations found related to gender [28]. Boys were more likely to overestimate their weight status compared to girls, who were more likely to underestimate it. Our findings can be supported by cultural differences and societal norms. In fact, in many emerging economy countries, particularly within Arab culture, thinness is often socially undesirable, with plumpness symbolizing fertility and womanhood [4]. Despite societal pressures on both genders to adhere to lean body ideals, girls appear to experience a higher degree of social and psychosocial stigma compared to boys [29].

However, in the past two decades, numerous developing countries have experienced extensive socio-cultural modernization, including the adoption of Western lifestyles emphasizing thinness as the ideal. This shift, amplified by social media, has altered cultural beliefs and beauty ideals. Consequently, many young people, influenced by societal pressures, have become increasingly concerned with conforming to a thin ideal body shape and size [8].

Our results, revealing a significant association between the accuracy of weight estimation and the mother's education level, are consistent with existing literature [30]. This underscores the influential role of maternal education in forming adolescents' perceptions of body image.

Our findings underscore the need for family-focused interventions addressing body image concerns among adolescents. Educating mothers on accurate weight recognition appears to be an effective strategy to correct adolescents’ weight misperceptions. Parents who fail to recognize and acknowledge overweight and obesity as health issues in a child are unlikely to seek professional medical advice or intervention. Addressing body weight misperceptions among adolescents, including those of normal weight, is crucial. This calls for school-based intervention strategies to rectify these misconceptions and educational workshops targeting adolescents about body awareness, especially addressing young girls regarding healthy body weight. Indeed, launching a national campaign to reshape social and cultural norms could act as an immediate signal, fostering societal acknowledgment and action on this critical issue. Implementing straightforward scales and benchmarks can aid adolescents in objectively assessing their weight, diverting from common societal, peer, or media-driven ideals.

This study had some limitations. Although our results align with those of previous studies in Tunisia, the sample is not nationally representative as it was confined to one urban area. Although cross-sectional studies are useful for characterizing the prevalence of a condition or a risk factor in a study population, their inability to demonstrate a temporal relationship limits the ability to infer causation. To address these issues and gain more generalizable insights, future research should encompass a diverse range of regions and possibly employ longitudinal study designs to observe changes and trends over time.

Our study has several strengths, with the primary one being its large and randomized sample population. Additionally, this research stands out as one of the few studies on this specific topic conducted in Tunisia. The use of objectively measured anthropometric measurements further enhances the reliability of our findings. There have been few studies that have compared perceived weight status and BMI status using measured weight and height. Most studies have relied on self-reported weight and height data, clearly posing some limitations on the accuracy of BMI calculations.

## Conclusions

Our study highlighted the magnitude of body weight misperception among Tunisian adolescents. Almost half of our study population underestimated their weight status, with over a quarter objectively being overweight or obese. This underscores the indisputable value of future research as well as preventive programs and informational campaigns directed at both adolescents and parents. Such campaigns are crucial to educate them on accurately evaluating their weight status and recognizing weights that pose health risks, guiding them to adopt measures to address any issues identified.
